# Materno-Fetal and Neonatal Complications of Diabetes in Pregnancy: A Retrospective Study †

**DOI:** 10.3390/jcm9092707

**Published:** 2020-08-21

**Authors:** Giampiero Capobianco, Alessandra Gulotta, Giulio Tupponi, Francesco Dessole, Maddalena Pola, Giuseppe Virdis, Marco Petrillo, Valerio Mais, Giorgio Olzai, Roberto Antonucci, Laura Saderi, Pier Luigi Cherchi, Salvatore Dessole, Giovanni Sotgiu

**Affiliations:** 1 Gynecologic and Obstetric Clinic, Department of Medical, Surgical and Experimental Sciences, University of Sassari, 07100 Sassari, Italy; alessandragulotta87@gmail.com (A.G.); giuliotup@hotmail.it (G.T.); francescodessole@gmail.com (F.D.); maddy.po@hotmail.it (M.P.); giuseppevirdis1@gmail.com (G.V.); marco.petrillo@gmail.com (M.P.); pcherchi@uniss.it (P.L.C.); dessole@uniss.it (S.D.); 2 Gynecologic and Obstetric Clinic, University of Cagliari, 09121 Cagliari, Italy; gineca.vmais@tiscali.it; 3 Neonatal Intensive Care Unit (NICU), Sassari University, 07100 Sassari, Italy; maurogiorgio.olzai@aousassari.it; 4 Pediatric Clinic, Sassari University, 07100 Sassari, Italy; rantonucci@uniss.it; 5 Clinical Epidemiology and Medical Statistics Unit, Department of Medical, Surgical and Experimental Sciences, University of Sassari, 07100 Sassari, Italy; lsaderi@uniss.it (L.S.); gsotgiu@uniss.it (G.S.)

**Keywords:** gestational diabetes mellitus (GDM), diabetes mellitus type 1 (DM1), diabetes mellitus type 2 (DM2), fetal outcomes, neonatal outcomes

## Abstract

The aim of this case–control study was to evaluate maternal–fetal and neonatal clinical outcomes in a group of patients with gestational diabetes mellitus (GDM) and pregestational diabetes such as diabetes mellitus type 1 (DM1) and diabetes mellitus type 2 (DM2) and compare them with those of patients without diabetes. A total of 414 pregnant women, nulliparous and multiparous, with single pregnancy were recruited. The selected patients were divided into two groups. Among 207 patients (group cases), 183 had GDM and 24 pregestational diabetes (of which *n* = 17 diagnosed with DM1 and *n* = 7 with diagnosis of DM2). Two-hundred-seven patients with a negative pathologic history of GDM, DM1 and DM2 represented the population of controls (group control). We reported an incidence of preterm delivery of 23.2% in the group of cases, of 18.3% in the group of patients with GDM and 66.7% in the group of patients DM1/2. Fetal growth disorders, such as intrauterine growth retardation (IUGR), small for gestational age (SGA), fetal macrosomia, were detected in four fetuses out of 207 (1.93%) in the control group and 20 fetuses out of 207 in the case group (9.67%, *p*-value 0.001); of these 16 of 183 fetuses of the GDM group (8.74%, *p*-value 0.002) and 4 of 24 fetuses of the DM1/2 group (16.67%, *p*-value 0.005). A very strong correlation between diabetes mellitus type 1 and preeclampsia (*p*-value < 0.0001) was observed. Close monitoring of pregnant women with diabetes is recommended to prevent maternal–fetal and neonatal complications.

## 1. Introduction

Gestational diabetes mellitus (GDM) is defined as a carbohydrate intolerance of variable severity with onset or first recognition in pregnancy [1]. The prevalence of diabetes in pregnancy has been increasing worldwide in parallel with obesity. The majority is gestational diabetes mellitus (GDM) with the remainder primarily pregestational diabetes (PGDM) such as pre-existing diabetes mellitus type 1 (DM1) and diabetes mellitus type 2 (DM2) [2].

The island of Sardinia, Italy, has an incidence rate of 33.4 per 100,000 of DM1 which is the second highest in the world after Finland [3]. Murgia et al. [4] reported a very high prevalence (22.3%) of GDM in a large group of Sardinian women in comparison to that of other Italian regions where it can reach up to 11% [5]. Shefali et al. [6] studied the effect of diabetes on pregnancy outcomes, comparing pregestational diabetes mellitus (PGDM; 79 women) and GDM (146 women) with non-diabetic mothers. They observed that abortions and low birth weight (<2500 g) were more common in the PGDM group than the GDM group.

Macrosomia, neonatal hypoglycemia, hyperbilirubinemia, shoulder dystocia, birth trauma and stillbirth could be maternal–fetal and neonatal complications of women with GDM [7,8,9,10]. Recent studies [11,12,13] have demonstrated an increased risk of congenital anomalies among women with GDM and especially with PGDM. In fact, Garne et al. [11] reported that multiple congenital anomalies were present in 13.6% of diabetes cases and 6.1% of non-diabetes cases. Parimi and Nitsch [12] demonstrated that offspring born to mothers with any form of diabetes in pregnancy had 50% increased risk of congenital anomalies of the kidney and the urinary tract (CAKUT). They concluded that 2.0% to 3.7% of cases of CAKUT in the USA could be eliminated if gestational diabetes were prevented or eliminated. Agha et al. [13] observed a 47% higher prevalence of congenital abnormalities overall and up to a three- to five-fold higher prevalence of various cardiac and CNS anomalies among newborns of mothers with diabetes compared with those born to mothers without diabetes.

The aim of our study was to evaluate maternal–fetal and neonatal clinical outcomes in a group of patients with gestational diabetes mellitus (GDM) and pregestational diabetes (DM1 and DM2) and compare them with those of patients without diabetes or other diseases potentially able to affect pregnancy. We also assessed whether different types of diabetes mellitus could be linked to different pregnancy and neonatal outcomes such as congenital anomalies.

## 2. Methods

In this retrospective study, 414 patients were recruited, nulliparous and multiparous, with single pregnancy, between 18 and 47 years old, who gave birth at the Obstetric and Gynecologic Clinic of Sassari University, Italy, in the period between January 2017 and December 2018.

The study was approved by the ethics committee of Sassari University in Italy.

The selected patients were divided into two groups:

- 207 patients (*group cases*): 183 had diagnosis of gestational diabetes mellitus and 24 pregestational (of which *n* = 17 diagnosed with diabetes mellitus type 1 and *n* = 7 with diagnosis of diabetes mellitus type 2);
- 207 patients (*group control*) with a negative pathologic history of GDM, DM1 and DM2 represented the population of controls (the control group was randomly selected to avoid selection bias).

The two groups were homogeneous by age (calculated at the time of delivery), with a ratio of 1:1.

The diagnosis of GDM in the cases included in the study was made using OGTT (oral glucose tolerance test) with 75 gr. of glucose (threshold values for the diagnosis of 92 mg/dL for fasting blood glucose, 180 mg/dL for blood glucose after one hour and 153 mg/dL after 2 h of glucose administration. Only one altered value was sufficient to diagnose GDM) between the 16th and 18th week in patients at high risk of GDM (body mass index, BMI > 30, GDM in the previous pregnancy, two blood glucose levels high at the first check) and between 24th and 28th week of pregnancy in medium risk patients [5,14]. The diagnosis of diabetes mellitus type 1 and type 2 in the cases submitted to our study had to be known before pregnancy.

We focused on the type of treatment performed in pregnancy by diabetic patients and how this affects the various outcomes analyzed. We also evaluated whether there was any relationship between the body mass index (BMI) of the patients examined and the risk of pregnancy complications.

All information regarding recruited patients, characteristics of pregnancy and delivery were collected by examining the admission and delivery records and the patient records. Neonatal data were collected from the admission registries and from the medical records of newborns admitted to the neonatal intensive care unit (NICU).

### 2.1. Collected Data

The data collected concerning the general characteristics of the patients were: age, parity, height, pregravidic weight, birth weight, last menstruation, possible comorbidity, performing prenatal screening surveys such as combined test, non invasive prenatal test (fetal DNA test), villocentesis, amniocentesis and fetal echocardiography. We also collected data on the type of treatment performed during pregnancy by diabetic patients: diet therapy or insulin.

The variables collected for each group of women were summarized in the following categories: pregnancy outcomes, diseases of pregnancy and fetal pathologies and neonatal outcomes.

### 2.2. Pregnancy Outcomes

- Gestational age at childbirth (GA)
- Number of hospitalization days
- Mode of delivery (spontaneous vaginal delivery or cesarean section)

### 2.3. In Case of Vaginal Delivery

- Spontaneous labor or induced labor
- Method of induction of labor for delivery (oxytocin or prostaglandins)
- Entities of blood loss, and therefore any postpartum hemorrhages (defined as all cases with loss greater than 500 mL of blood)
- External genital conditions after delivery: genital lacerations or episiotomy.

### 2.4. In Case of Cesarean Section (CS)

- Scheduled or emergency cesarean section
- Indications for surgery
- Entities of blood loss, and therefore any postpartum hemorrhages (defined as all cases with loss greater than 1000 mL of blood).

### 2.5. Diseases of Pregnancy and Fetal Pathologies

- Threatened abortion
- Threatened preterm birth
- Gestational hypertension
- Preeclampsia and hemolysis, elevated liver enzymes, low platelets (HELLP) syndrome
- Placental abruption
- Pathology of amniotic fluid (oligohydramnios and polydramnios)
- Premature rupture of membranes (PROM)
- Macrosomia
- Intrauterine growth retardation (IUGR) fetus
- Morphologic abnormalities diagnosed on ultrasound

### 2.6. Neonatal Outcomes

- Weight at birth compared to those expected for the gestational age (in percentiles) and then classification within one of the classes of appropriate for gestational age (AGA), small for gestational age (SGA), or or large for gestational age (LGA). For this study we used the definition of the Royal College of Obstetricians and Gynecologists (RCOG) [15] which informs UK clinical practice, based on sonographic estimated fetal weight (EFW) measurement < 10th percentile to describe a fetus that has not reached its target weight. Patients were divided in three groups for comparison; fetuses with EFW below the 10th percentile for gestational age (SGA), fetuses with EFW > 10th percentile for gestation (AGA) and fetuses > 90th percentile for gestation (LGA) according to the Alexander growth standard [16].
- Apgar at the first minute
- Number of hospitalization days and at which intensity of care (nursery, neonatology or NICU)
- Recognition of respiratory diseases at birth such as respiratory distress syndrome (RDS), transient tachycardia of the newborn (TTN) or apnea crisis and if there was any intubation
- Blood glucose at the third hour
- Hypoglycemia status and glucose supplementation
- Neonatal jaundice, treated or not with phototherapy
- Morphologic abnormalities found at birth

### 2.7. Statistical Analysis

Qualitative and quantitative variables were collected using an ad hoc Excel^®^ (Microsoft^®^, www.microsoft.com, Redmond, WA, USA) spreadsheet. The former were described with absolute and relative (percentages) frequencies, whereas the latter with means (standard deviations, SD) or medians (interquartile ranges, IQR) depending on their normal or non-normal distribution, respectively. chi-squared or Fisher’s exact test were used to compare individuals with and without diabetes for qualitative variables, whereas Student’s t or Mann–Whitney test for normal or non-normal quantitative variables, respectively. univariate logistic regression analysis was performed to assess the relationship between pregnancy and fetal complications and clinical (e.g., diabetes), epidemiological and demographic variables. A two-tailed *p*-value < 0.05 was considered statistically significant. Statistical software STATA version 15 (StataCorp, 2019: Release 16. College Station, TX, USA) was adopted for all statistical analyses.

## 3. Results

A significantly higher pregestational body weight (median (IQR): 65 (58–80) VS. 56 (50–64), *p*-value < 0.0001) and pregestational BMI (median (IQR) 25.6 (22.5–30.5) VS. 22.0 (19.8–24.0), *p*-value < 0.0001) were detected in patients with GDM and DM1/DM2 in comparison those in the control group. Prevalence of obese patients (25.6%) was significantly higher in the cases if compared with the prevalence of the control group (3.9%) (*p*-value < 0.0001). The median (IQR) weight gain in pregnancy was shown to be higher in the control group (12 (10–15) (12 kg) compared to case group (10 (6–13)) (*p*-value < 0.0001). A significant excessive weight increase during pregnancy was found to be more prevalent in the control group (40.6%) than in the case group (25.6%) (*p*-value = 0.001); A significant higher body weight and BMI were detected at the time of delivery in the group of cases when compared with the control group (Table 1).

### 3.1. Pregnancy Disorders

There was no statistically significant difference between case and control groups for the following outcomes: threatened abortion, anomalies of placental insertion and placental abruption, pathologies of the amniotic fluid (oligohydramnios and polydramnios), gestational hypertension, preeclampsia, HELLP syndrome and PROM.

A statistically significant difference in the frequency of threatened preterm birth was reported in 35 patients of 207 in the case group (16.9%) in comparison with 15 of 207 patients in the control group (7.3%) (*p*-value = 0.003). This difference was more evident when considering the incidence of preterm delivery in patients with GDM (27 patients of 183, 15.4% *p*-value 0.01) and DM1/DM2 (8 patients of 24, 33.3% *p*-value < 0.0001).

### 3.2. Fetal Disorders

There was a statistically significant difference in fetal growth disorders such as: IUGR fetus, SGA fetus or fetal macrosomia. Fetal growth disorders were detected in 4 fetuses (1.9%) in the control group and 20 in the case group (9.7%, *p*-value 0.001). The increased incidence of fetal macrosomia (fetal growth ≥ 95° percentile) was found in 10 fetuses from diabetic mothers (*p*-value: 0.002): 7 in the GDM group (*p*-value 0.004) and 3 in the DM1/2 group (*p*-value: 0.001).

Fetal echocardiography was used to investigate cardiac abnormalities more frequently in the group of cases (32% VS. 14%; *p*-value < 0.0001).

### 3.3. Pregnancy Outcomes

A statistically significant difference in the CS delivery between case and control groups were recorded (126, 60.9% VS. 86, 41.5%, *p*-value < 0.0001). This finding was confirmed considering the GDM group (104 CS on 183 delivery, 56.8%, *p*-value 0.001) and DM1/2 group (22 CS on 24 delivery, 91.6%, *p*-value < 0.0001).

CS in emergency was more prevalent in the DM1/2 group (63.6%, 14 urgent CS of 22) when compared with that in control group (39.5%, 34 urgent CS of 86), (*p*-value 0.04).

Medical induction of vaginal delivery in the group of diabetic patients was more frequent than in the control group (24/81, 29.6%; *p*-value < 0.0001).

Preterm deliveries (48 (23.2%) VS. 14 (6.8%), *p*-value < 0.0001) were more frequent in the case group.

### 3.4. Comparison between Pathologies in Pregnancy and Diabetes Therapy

51.7% (107/207) cases received insulin therapy (injective or through a pump), whereas 48.3% received a diet-behavioral therapy. No statistically significant differences were found, with the only exception of an increased incidence of threatened preterm birth in patients receiving insulin therapy (21.5% VS. 11%; *p*-value: 0.048); and an increased risk of premature rupture of membranes at the end of pregnancy, in patients on diet therapy (18% VS. 3.7%; *p*-value: 0.001).

### 3.5. Relationship between Pregnancy Complications and a BMI > 25 in Cases and Controls

The logistic regression analysis performed showed no statistically significant association between a BMI > 25 and pregnancy complications.

### 3.6. Relationship between Materno-Fetal Outcomes and Forms of Diabetes 

DM1 was significantly associated with threatened preterm birth (OR (95% CI): 4.1 (1.4–11.5); *p*-value = 0.0009), preeclampsia (OR (95% CI): 10.9 (3.0–39.5); *p*-value < 0.0001), preterm birth (OR (95% CI): 10.3 (3.4–31.0); *p*-value < 0.0001) and recourse to CS as a way to perform delivery (OR (95% CI): 11.6 (1.5–89.6); *p*-value = 0.02). diabetes mellitus type 2 was associated with threatened abortion (OR (95% CI): 79.6 (6.2–1029); *p*-value = 0.001). GDM was associated with a minor risk of threatened abortion (OR (95% CI): 0.1 (0.0–0.7); *p*-value = 0.03), threatened preterm birth (OR (95% CI): 0.4 (0.2–0.9); *p*-value = 0.04), gestational hypertension (OR (95% CI): 0.2 (0.0–0.8); *p*-value = 0.02), preeclampsia (*p*-value 0.004), preterm birth (OR (95% CI): 0.1 (0.0–0.3); *p*-value < 0.0001) and recourse to CS (OR (95% CI): 0.1 (0.0–0.5); *p*-value = 0.004) (Table 2).

### 3.7. Neonatal Outcomes

The median length of hospital stay was 3 days for the births of the control group, against 4 days for that of the GDM group, (*p*-value < 0.0001) and 10 days for that of the DM1/2 group (*p*-value < 0.0001).

Twenty-one controls were admitted to neonatal intensive care unit (NICU), compared with 69 out in the case group (*p*-value < 0.0001), 26.9% of the births of the GDM group and 79.2% of the births of the DM1/2 group (*p*-value < 0.0001). 8.7% of the births of the control group showed respiratory disorders against 17.9% of the births of the case group (*p*-value: 0.004), 15.8% of births of the GDM group (*p*-value 0.03) and 33.3% of births of the DM1/2 group (*p*-value < 0.0001).

Respiratory distress syndrome (RDS) occurred in 5.3% of the births of the control group against 12.6% of the births in the case group. Incidence of RDS was higher in neonates of the DM1/2 group (29.2%, *p*-value < 0.0001).

Infants requiring intubation were 12 in the control group and 31 in the case group.

There were 11 newborns with neonatal hypoglycemia in the control group against 51 newborns in the case group (*p*-value: 0.0001)

Incidence of neonatal jaundice was 51.7% 23.7% in the case and control group (*p*-value < 0.0001), respectively.

Morphologic anomalies were detected in 20 (9.6%) neonates in the control group and 40 (19.3%) in the case group (*p*-value: 0.005) (Table 3).

Specifically, in the group of cases, the following anomalies were identified:

Cardiovascular system:

- 3 cases of patent foramen ovale with hemodynamically significant shunt;
- 3 cases of paroxysmal supraventricular tachycardia (PST);
- 1 case of atrial septal defect (ASD) type “ostium secundum”, associated with patency of the ductus arteriosus;
- 1 case of ventricular septal defect (VSD) with left–right shunt;
- 1 case of aortic coarctation;
- 1 case of right bundle branch block, associated with severe laryngomalacia.

Musculoskeletal system:

- 4 cases of congenital clubfoot or talipes equinovarus;
- 3 cases of clinodactyly in the 5th right toe;
- 1 case of mandibular hypoplasia with lingual retropulsion, associated with the presence of hammer toes.

Urogenital apparatus:

- 5 cases of hydrocele;
- 5 cases of bilateral pyelectasis;
- 1 case of pelvic kidney;
- 1 case of hypospadias;
- 1 case with hypertrophy of labia minora;
- 1 case with left ovarian mass (21 mm x 18 mm).

Central nervous system:

- 1 case of hypoplasia of the cerebellar vermis with concomitant enlargement of the cavity of the 4th ventricle and increased volume of the cisterna magna.

Genetic defects:

- 1 case with Robertsonian translocation between chromosomes 13–14 of maternal origin.

Other anomalies: 3 cases of short lingual frenulum, 1 case of umbilical hernia, 1 case of cleft palate.

The following anomalies were found in the control group:

Cardiovascular system:

- 1 case of ventricular septal defect (VSD), with the presence of a shunt of medium size;
- 1 case of mid-apical VSD with the presence of a mild shunt;
- 1 case of patency of the ductus arteriosus.

Musculoskeletal system:

- 1 cases of congenital clubfoot or talipes equinovarus;
- 1 case of mandibular hypoplasia;
- 1 case of clinodactyly in the 5th toe of the left foot;
- 1 case of mandibular hypoplasia associated with clinodactyly.

Uro-genital apparatus:

- 2 cases of hypospadias;
- 1 case of hydrocele;
- 1 case of renal pyelectasis left;

Genetic defects:

- 1 case of trisomy 21 (Down’s syndrome);
- 1 case of a newborn with facial asymmetry, left ear dysmorphism, absence of the last pair of ribs, patent foramen ovale and retinal hemorrhages, which posed the suspicion of Goldenhar syndrome.

Other anomalies: 1 case of short lingual frenulum; 2 cases of umbilical hernia.

## 4. Discussion

In the present study, the diabetic patients belonging to the case group and the patients belonging to the control group had different pregravidic anthropometric characteristics. Patients with GDM had a median value of the pregravidic body weight higher than that of the patients in the control group and the median pregravidic BMI differed by 3.6 points between two groups (25.6 in the group of cases and 22 for the control group). This difference had the same degree of significance regardless of the type of diabetes considered, GDM or DM type 1 and 2.

An Australian study showed that the prevalence of GDM tends to increase in relation to the BMI categories considered, it affects 6.74% of overweight patients, 13.42% of patients with mild obesity of grade 1, 12.79% of patients with moderate obesity of Grade 2 and 20% of patients with severe obesity of grade 3 [17]. In our study, the weight gain in pregnancy was greater in the patients belonging to the control group (median 12 kg) compared to the patients of the case group (median 10 kg). It was also found that patients in the control group were more frequently faced with an excessive weight increase in pregnancy compared to diabetic patients (40.6% versus 25.6%). Some studies have shown that the increase of the pregravidic BMI correspond to a lower weight gain during pregnancy, probably linked to greater dietary and behavioral control to which patients are subjected, when risk factors or metabolic pathology is already known [18]. However, BMI at childbirth was significantly higher in diabetic patients (median 29.4 cases vs. 26.7 controls), the dietary–behavioral control in these patients was apparently not sufficient to reverse the differences with the patients in the control group. We reported no relationship between the incidence of pregnancy complications such as the threatened abortion, threatened preterm delivery, gestational hypertension, preeclampsia, placental abruption, the quantitative alteration of amniotic fluid, PROM and elevated BMI in diabetic patients.

Conflicting data exist in the literature. In fact, according to Martin et al. [17], the association of GDM and BMI > 25 did not determine any statistically significant risk of incurring obstetric complications compared to that determined by these individually taken variables. The only statistically significant difference is related to the gestational age at childbirth (average of 38 weeks), which in obese patients with GDM differs by 2.3 standard deviations compared to that of patients with only diabetes mellitus or only high BMI (obese or overweight) or association with diabetes and overweight status. According to Sugiyama et al. [18] instead, the association between GDM and high BMI seemed to predispose to several obstetric complications. In fact, they reported: pregnancy induced hypertension (PIH) (1.5% of cases in the control group, versus 10.8% in patients with GDM and BMI between 25 and 30, and 14.8% of patients with GDM and BMI > 30), the need for emergency cesarean section (incidence of 28.1% in the control group, compared with 39.2% of patients with GDM and BMI between 25 and 30 and 46.2% of patients with GDM and BMI > 30), the use of primary scheduled caesarean section (9.9% of cases in the group of control, against 21.8% of patients with GDM and BMI between 25 and 30 and 29.3% of those with GDM and BMI > 30), and finally the need of medical induction of labor (21.9% of patients in the control group, against 24.2% of those with GDM and BMI between 25 and 30 and 30.4% of those with GDM and BMI > 30).

With regard to the risk of miscarriage in diabetic patients we showed no statistical significance among 3 of 207 diabetic patients (1.5%) and 4 of 207 in the control group (1.9%). However, the increased risk of spontaneous miscarriage in diabetic patients is well documented in the literature, but our result may be affected by the sample. In fact, we included in our study only pregnancies that resulted in a term or preterm delivery, excluding miscarriages and intrauterine fetal death. Evaluating data on the DM1/2 patient group, it is interesting to note that the threatened abortion affected 2 patients of 24 (8.3% of cases versus 1.9% of controls), although the literature data showed that the rate of miscarriages in DM type 1 and DM type 2 were 2.6% and 3.7%, respectively [19]: in this case, however, given the small sample considered, it is not possible to achieve statistical significance.

By performing a logistic regression analysis between the various types of diabetes and the various complications considered, an association emerged between the threatened abortion and diabetes mellitus type 1 (*p* value 0.001), and to a lesser extent also with GDM (*p* value 0.05). Other conditions for which no statistically significant data have emerged were placental pathologies, oligohydramnios and polyhydramnios and PROM at the end of pregnancy (27 cases in the control group and 22 among the diabetics: 13% versus 10.6% of total) or preterm (4 patients in the control group against 6 diabetics: 1.9% vs. 2.9%). However, the correlation between GDM and threatened preterm birth and preterm delivery were statistically significant. Preterm delivery may occur by spontaneous onset or may be induced by the need to schedule childbirth, to prevent maternal and/or fetal complications and to reduce the risk of perinatal mortality. We reported an incidence of preterm delivery of 23.2% in the group of cases, of 18.3% in the group of patients with GDM and 66.7% in the case group DM1/2; the most important causes of preterm childbirth were uterine overdistension due to fetal macrosomia and/or polyhydramnios, PROM. Dollberg et al. [20] did not associate the high incidence of preterm childbirth with polyhydramnios but recognized as a risk factor the presence of genitourinary infections and a positive history for previous preterm deliveries.

Gestational hypertension (GH), preeclampsia and HELLP syndrome are complications that we considered in the case of diabetic patients alone, avoiding the inclusion in the control group of patients presenting with one or more of these clinical conditions, so as to eliminate any confounding factors on the remaining maternal–fetal outcomes taken into consideration. From our study it emerged that there were 7 GH cases in total (3.4%) with 4 in the GDM group (2.19%) and 3 in the group with pregestational diabetes (12.5%). There were 12 cases of preeclampsia in total (5.8% of cases), with 7 patients affected by the GDM group (4%) and 5 in the group with pregestational diabetes (20.8%). There were 2 cases of HELLP syndrome (1% of cases), all in patients with GDM. Both patients were subjected to emergency preterm cesarean section after the onset of preeclampsia (29th week of pregnancy in the first case and 33rd week in the second case). HELLP syndrome occurred in both cases on the first day of puerperium, requiring the admission of both patients to the intensive care unit of the University Hospital. The relationship between these complications and the various forms of diabetes showed a very strong correlation between diabetes mellitus type 1 and preeclampsia (*p*-value < 0.0001), while the relationship between this and GDM was less pronounced, but still statistically significant (*p*-value 0.004). Another interesting fact is that in all three types of diabetes there was a statistically significant increase in the incidence of preterm delivery.

As far as fetal outcomes are concerned, we have focused mainly on the evaluation of intrauterine fetal growth pathologies such as fetal macrosomia and IUGR. Ten of 207 (4.8%) fetuses in the case group were IUGR and 4 of 207 in the control group (1.9%), so there seemed to be a statistically significant difference between the group of diabetic patients and the control group. No cases of macrosomia were recorded in the control group, compared with 10 cases of 207 in the case group (10.8%), 7 macrosomal fetuses in 183 pregnant patients with GDM (4%) and 3 of 24 pregnant patients with DM1/2 (12.5%). 22.3% of those born in the case group were large for gestational age (LGA) infants compared to 9% of the control group. The data of the literature is comparable to those obtained by us. A Swedish study [21] has in fact studied the characteristics of newborns of women with GDM between 1998 and 2007, for a total of 1547 infants, comparing them with those of a control group composed of over 83,000 infants of non-diabetic mother; the incidence of LGA births was 26% in children of diabetic mothers and 10.6% in those of the control group.

An interesting fact emerging from our study concerns the developmental percentiles of the fetus, where newborns of pregestational diabetes women had a median value equal to 88° percentile. These data were indicative of the fact that fetal macrosomia was most closely linked to type 1 and type 2 diabetes compared to gestational diabetes (which in its median value differed by 14 percentile points from the data attributable to births from non-diabetic patients).

With regard to the mode of delivery, our results are overlapping with those present in the literature. There was a strong tendency, in diabetic patients to perform the delivery by cesarean section (in our study the values were 58.5% of CS for the case group and 41.6% of CS in the control group). This is all the more evident considering the only patients with DM1/2, for whom a percentage of cesarean sections of 91.7% emerged, which was carried out in emergency mode in 63.6% of the cases, against 39.5% of emergency CS in the control group. The difference did not appear statistically significant in the comparison between the global case group and the group of controls. In the literature, a recent study demonstrated that there is a significant difference between cesarean section performed in emergency mode in patients with GDM compared to the control group (31.6% vs. 19.4%) [22]. This evidence, though statistically significant, differed from that observed in our study.

In our study, we reported a statistically significant difference between the group of cases and controls in the use of medical induction to labor. By the use of prostaglandins or oxytocin, depending on the Bishop’s score presented, 24 of 81 (29.6%) were induced in the case group and 8 of 121 (6.6%) in the control group. This is consistent with the fact that diabetic patients are often subjected to medical induction of labor at a gestational age that represents a good compromise between the survival of the newborn and avoidance of complications related to the continuation of pregnancy in an unfavorable metabolic context.

As regards the relationship between antidiabetic therapy and pregnancy pathologies, our study highlighted an increased incidence of threatened preterm delivery in patients on insulin therapy, while patients using dietetic therapy showed a greater risk of incurring PROM at term of pregnancy. All other outcomes analyzed were homogeneous among patients using insulin therapy and patients using dietetic therapy. According to a study of Benhalima et al. [23], insulin therapy in pregnancy was not able to prevent complications related to metabolic pathology such as the higher incidence of LGA infants (28.5% vs. 13.1%, *p*-value < 0.0001) and greater recourse to CS (44.1% vs. 27%, *p*-value 0.001).

A different incidence of morphologic anomalies emerged from our study, depending on whether the study was carried out on the fetus using ultrasound or on the newborn. In fact, according to fetal ultrasonographic study, there was no statistically significant difference in the incidence of morphologic anomalies found in the case group and that of controls. However, in the post-partum period, the direct observation of the newborn allowed identification of a much greater number of morphologic anomalies among those newborns from diabetic patients compared to the births of the control group. The reason for this apparent incongruity was related to the fact that the dysmorphic features highlighted were largely represented by anomalies in the body structures that were not detected by ultrasound (i.e., clinodactyly, short lingual frenulum, hypospadias). Thus, so many anomalies, which were well tolerated and compatible with life, could only be highlighted at birth. All the anomalies that we detected are well documented in literature in association with diabetes.

Among the neonatal outcomes that we analyzed, the variables linked to respiratory disorders at birth seemed to differ from the data present in the literature [24,25,26]. The incidence of TTN in our study was 1.5% in the control group and 1.9% in the births of the case group, showing no statistically significant difference; in literature, this percentage among diabetic newborns was greater, around 10%; the data related to respiratory distress syndrome (RDS) showed greater incidences in our study than in the literature [26].

An incidence of 25.1% of neonatal hypoglycemia in births from diabetic mother in accordance with the data present in the literature had been highlighted and the risk of incurring this complication was greater in infants of mothers with type 1 diabetes (58.3%) [27]. The percentage of neonatal jaundice in the children of diabetic mothers ranged from 11% to 29%, depending on the case studies in the literature [26,27]. Our study revealed a higher incidence compared to these data (51.7%) but considering only the cases of jaundice that needed phototherapy the data decreased considerably (32.8%), very close to the information in the literature mentioned above.

### Limitations of the Study

The number of women with pregestational diabetes (*n* = 24) was very low to make meaningful conclusions. The retrospective nature of the study and the poor sample size can hinder the statistical inference. However, they represent the epidemiological perspective of an Italian regional area. Our conclusion stated that there was “a very strong correlation between diabetes mellitus type 1 and preeclampsia”. This statement is perhaps too strong since there are only 7 patients with type 1 diabetes in their cohort. A small sample cannot support generalizations. Thus, further studies with larger sample size are needed to draw definitive conclusions.

## 5. Conclusions

It is of fundamental importance to identify at risk patients in advance, so that rigorous preventive measures can be put in place.

It is also important to identify women at risk of pregestational diabetes prior to conception, to avoid the onset of GDM in non diabetic patients by educating them to correct modifiable risk factors (e.g., sedentary lifestyle, weight, nutrition).

Glycemic control in patients with pregestational diabetes is recommended, so as to reduce the occurrence of maternal–fetal complications and neonatal adverse events. It is, therefore, recommended to intensify the strategies of sensitization using different figures involved in the care of these patients (gynecologists, diabetologists, nutritionists and all healthcare professionals), and by making patients aware of the fact that planning pregnancy with normal glucose levels of HbA1c under 6.5% significantly reduces the risk of experiencing adverse fetal and maternal events. However, with regard to prediabetes the severity of adverse outcomes lies between PGDM and GDM developed during pregnancy.

Good glycemic control and therefore a pregnancy with fewer risk factors reduces the incidence of operative delivery in favor of the physiological vaginal delivery and reduces the risk of preterm birth, which has negative influences on neonatal outcomes.

In summary, patients with GDM had a median value of the pregravidic body weight higher than that of the patients in the control group and the median pregravidic BMI differed by 3.6 points between two groups (25.6 in the group of cases and 22 for the control group). This difference had the same degree of significance regardless of the type of diabetes considered, GDM or DM type 1 and 2. We reported no relationship between the incidence of pregnancy complications such as the threatened abortion, threatened preterm delivery, gestational hypertension, preeclampsia, placental abruption, the quantitative alteration of amniotic fluid, PROM and elevated BMI in diabetic patients. By performing a logistic regression analysis between the various types of diabetes and the various complications considered, an association emerged between the threatened abortion and diabetes mellitus type 1 (*p*-value 0.001), and to a lesser extent also with GDM (*p*-value 0.05). Fetal growth disorders, such as intrauterine growth retardation (IUGR), small for gestational age (SGA), fetal macrosomia, were detected in 4 fetuses of 207 (1.93%) in the control group and 20 fetuses of 207 in the case group (9.67%, *p*-value 0.001); of these 16 of 183 fetuses of the GDM group (8.74%, *p*-value 0.002) and 4 of 24 fetuses of the DM1/2 group (16.67%, *p*-value 0.005). The correlation between GDM and threatened preterm birth and preterm delivery were statistically significant. A very strong correlation between diabetes mellitus type 1 and preeclampsia (*p*-value < 0.0001) was observed. The dysmorphic features highlighted were largely represented by anomalies in the body structures that were not detected by ultrasound (i.e., clinodactyly, short lingual frenulum, hypospadias).

## Figures and Tables

**Table 1:** Anthropometric characteristics of the patients.

**Anthropometric Characteristics**	**Control Group** **(*n* = 207)**	**Case Group** **(*n* = 207)**	***p*-Value**
Median (IQR) pregestational weight, kg	56 (50–64)	65 (58–80)	<0.0001
Median (IQR) pregestational BMI, kg/m^2^	22.0 (19.8–24.0)	25.6 (22.5–30.1)	<0.0001
Normal weight, *n* (%)	175 (84.5)	92 (44.4)	<0.0001
Overweight, *n* (%)	24 (11.6)	60 (29.0)	<0.0001
Obese, *n* (%)	8 (3.9)	53 (25.6)	<0.0001
Median (IQR) weight increase, kg	12 (10–15)	10 (6–13)	<0.0001
Excessive weight increase (>12 kg), *n* (%)	84 (40.6)	53 (25.6)	0.001
Median (IQR) weight at delivery, kg	70 (63–76)	78 (68–89)	<0.0001
Median (IQR) BMI at delivery, kg/m^2^	26.7 (24.7–28.7)	29.4 (26.6–33.1)	<0.0001
**Anthropometric Characteristics**	**Control Group** **(*n* = 207)**	**Women with GDM** **(*n* = 182)**	***p*-Value**
Median (IQR) pregestational weight, kg	56 (50–64)	65 (58–79)	<0.0001
Median (IQR) pregestational BMI, kg/m^2^	22.0 (19.8–24.0)	25.5 (22.6–30.1)	<0.0001
Normal weight, *n* (%)	175 (84.5)	83 (45.6)	<0.0001
Overweight, *n* (%)	24 (11.6)	53 (29.1)	<0.0001
Obese, *n* (%)	8 (3.9)	46 (25.3)	<0.0001
Median (IQR) weight increase, kg	12 (10–15)	10 (6–12)	<0.0001
Excessive weight increase (>12 kg), *n* (%)	84 (40.6)	46 (25.3)	0.001
Median (IQR) weight at delivery, kg	70 (63–76)	77 (68–88)	<0.0001
Median (IQR) BMI at delivery, kg/m^2^	26.7 (24.7–28.7)	29.5 (26.8–32.9)	<0.0001
**Anthropometric Characteristics**	**Control Group** **(*n* = 207)**	**Women with DM1/2** **(*n* = 24)**	***p*-Value**
Median (IQR) pregestational weight, kg	56 (50–64)	67.5 (57.5–81.0)	0.0004
Median (IQR) pregestational BMI, kg/m^2^	22.0 (19.8–24.0)	25.7 (21.2–30.1)	0.0005
Normal weight, *n* (%)	175 (84.5)	9 (37.5)	<0.0001
Overweight, *n* (%)	24 (11.6)	7 (29.2)	0.02
Obese, *n* (%)	8 (3.9)	6 (25.0)	<0.0001
Median (IQR) weight increase, kg	12 (10–15)	9.5 (6–13)	0.008
Excessive weight increase (>12 kg), *n* (%)	84 (40.6)	7 (29.2)	0.28
Median (IQR) weight at delivery, kg	70 (63–76)	79.5 (65.0–88.5)	0.006
Median (IQR) BMI at delivery, kg/m^2^	26.7 (24.7–28.7)	29.1 (25.7–33.6)	0.01

IQR—interquartile ranges; DM1—diabetes mellitus type 1; DM2—diabetes mellitus type 2; GDM—gestational diabetes mellitus; BMI— body mass index; normal weight—BMI between 18 and 24.9 kg/m^2^; overweight—BMI between 25 and 29.9 kg/m^2^; obese—BMI ≥ 30 kg/m^2^.

**Table 2:** Maternal-fetal outcomes and the various types of diabetes.

Pathologies in Pregnancy	OR (95% CI)		*p*-Value
Threatened abortion	DM1	–	–
	DM2	79.6 (6.2–1028.9)	0.001
	GDM	0.1 (0.0–0.7)	0.03
Threatened preterm birth	DM1	4.1 (1.4–11.5)	0.009
	DM2	0.8 (0.1–7.0)	0.85
	GDM	0.4 (0.2–0.9)	0.04
Gestational hypertension	DM1	4.9 (0.9–27.6)	0.07
	DM2	5.4 (0.6–52.0)	0.15
	GDM	0.2 (0.0–0.8)	0.02
Preeclampsia	DM1	10.9 (3.0–39.5)	<0.0001
	DM2	–	–
	GDM	0.2 (0.1–0.6)	0.004
HELLP syndrome	DM1	–	–
	DM2	–	–
	GDM	–	–
Placental abruption	DM1	–	–
	DM2	–	–
	GDM	–	–
Amniotic fluid pathology	DM1	3.9 (1.0–15.6)	0.06
	DM2	–	–
	GDM	0.4 (0.1–1.7)	0.22
Oligohydramnios	DM1	2.9 (0.3–27.6)	0.35
	DM2	–	–
	GDM	0.5 (0.1–5.0)	0.59
Polyhydramnios	DM1	4.1 (0.8–22.0)	0.10
	DM2	–	–
	GDM	0.4 (0.1–2.1)	0.27
Premature rupture of amniochorionic membranes—preterm	DM1	–	–
	DM2	6.5 (0.7–64.6)	0.11
	GDM	0.7 (0.1–6.1)	0.73
Premature rupture of amniochorionic membranes—to term	DM1	–	–
	DM2	–	–
	GDM	–	–
**Fetal pathologies**			
Fetal growth disorders	DM1	2.2 (0.6–8.4)	0.26
	DM2	1.6 (0.2–13.9)	0.68
	GDM	0.5 (0.2–1.7)	0.26
Macrosomia	DM1	3.0 (0.6–15.6)	0.18
	DM2	3.5 (0.4–32.6)	0.27
	GDM	0.3 (0.1–1.2)	0.09
IUGR	DM1	1.3 (0.2–10.6)	0.83
	DM2	–	–
	GDM	1.3 (0.2–10.3)	0.84
Morphologic anomalies	DM1	2.7 (0.5–13.6)	0.23
	DM2	–	–
	GDM	0.6 (0.1–2.9)	0.53
**Delivery**			
Vaginal delivery	DM1	0.1 (0.0–0.7)	0.02
	DM2	0.3 (0.0–2.1)	0.20
	GDM	8.8 (2.0–38.5)	0.004
Cesarean section	DM1	11.6 (1.5–89.6)	0.02
	DM2	4.0 (0.5–33.9)	0.20
	GDM	0.1 (0.0–0.5)	0.004
Preterm delivery	DM1	10.3 (3.4–31.0)	<0.0001
	DM2	4.7 (1.0–21.9)	0.05
	GDM	0.1 (0.1–0.3)	<0.0001
Hospitalization time ≥ 4 days	DM1	–	–
	DM2	–	–
	GDM	–	–

DM1—diabetes mellitus type 1; DM2—diabetes mellitus type 2; GDM—gestational diabetes mellitus; HELLP—hemolysis, elevated liver enzymes, low platelets; IUGR—intrauterine growth retardation.

**Table 3:** Neonatal outcomes.

	**Control Group** **(*n* = 207)**	**Case Group** **(*n* = 207)**	***p*-Value**
Fetus large for gestational age (LGA) *n* (%)	18/207 (8.7)	46/207 (22.3)	<0.0001
Respiratory disorders *n* (%)	18/207 (8.7)	37/207 (17.9)	0.004
RDS (respiratory distress syndrome) *n* (%)	11/207 (5.3)	26/207 (12.6)	ns
TTN (Neonatal transient tachypnea) *n* (%)	3/207 (1.4)	9/207 (4.3)	ns
Neonatal intubation *n* (%)	12/207 (5.8)	31/207 (15)	ns
Median (IQR) glycemia at 3° hours, mg/dL	68 (61–74)	64 (54–73)	0.008
Median (IQR) lower glycemia, mg/dL	64 (54–71)	51 (39–62)	<0.0001
Neonatal hypoglycemia *n* (%)	11/207 (5.3)	51/207 (24.6)	0.0001
Neonatal jaundice *n* (%)	49/207 (23.7)	107/207 (51.7)	<0.0001
Phototherapy *n* (%)	24/207 (11.6)	68/207 (32.8)	<0.0001
Morphologic anomalies *n* (%)	20/207 (9.6)	40/207 (19.3)	0.005
	**Control Group** **(*n* = 207)**	**Women with GDM** **(*n* = 182)**	
Fetus large for gestational age (LGA) *n* (%)	18/207 (8.7)	34/182 (18.8)	<0.0001
Respiratory disorders *n* (%)	18/207 (8.6)	28/182 (15.4)	0.03
RDS (respiratory distress syndrome) *n* (%)	11/207 (5.3)	19/182 (10.4)	ns
TTN (Neonatal transient tachypnea) *n* (%)	3/207 (1.4)	7/182 (3.9)	ns
Neonatal intubation *n* (%)	12/207 (5.8)	22/182 (12.1)	0.03
Median (IQR) glycemia at 3° hours, mg/dL	68 (61–74)	65 (57–74)	ns
Median (IQR) lower glycemia, mg/dL	64 (54–71)	53 (42–63)	<0.0001
Neonatal hypoglycemia *n* (%)	11/207 (5.3)	37/182 (20.3)	0.0001
Morphologic anomalies *n* (%)	20/207 (9.6)	34/182 (18.8)	0.01
	**Control Group** **(*n* = 207)**	**Women with DM1/2** **(*n* = 24)**	
Fetus large for gestational age (LGA) *n* (%)	18/207 (8.7)	12/24 (50)	<0.0001
Respiratory disorders *n* (%)	18/207 (8.6)	8/24 (33.3)	<0.0001
RDS (respiratory distress syndrome) *n* (%)	11/207 (5.3)	7/24 (29.2%)	0.0001
TTN (neonatal transient tachypnea) *n* (%)	3/207 (1.4)	2/24 (8.3)	ns
Neonatal intubation *n* (%)	12/207 (5.8)	8/24 (33.3)	<0.0001
Median (IQR) glycemia at 3° hours, mg/dL	68 (61–74)	47.5 (31.0–66.5)	<0.0001
Median (IQR) lower glycemia, mg/dL	64 (54–71)	38.5 (29.5–51.5)	<0.0001
Neonatal hypoglycemia *n* (%)	11/207 (5.3)	14/24 (58.3)	0.0001
Morphologic anomalies *n* (%)	20/207 (9.6)	6/24 (25)	0.02
